# Expansion of Child Tax Credits and Mental Health of Parents With Low Income in 2021

**DOI:** 10.1001/jamanetworkopen.2023.56419

**Published:** 2024-02-21

**Authors:** Jaehyun Nam, Sarah Jiyoon Kwon

**Affiliations:** 1 Department of Social Welfare, Pusan National University, Busan, South Korea; 2 Crown Family School of Social Work, Policy and Practice, University of Chicago, Chicago, Illinois

## Abstract

**Importance:**

The 2021 Child Tax Credit (CTC) expansion, as part of the American Rescue Plan Act, offered financial relief through generous monthly tax benefits to families with children amid the COVID-19 pandemic. In light of heightened concerns about mental health during the pandemic, the expanded CTC may have alleviated parental mental health challenges, especially within families with low income.

**Objective:**

To investigate the association between the 2021 CTC expansion and mental health among parents with low income as measured by depression and anxiety symptoms.

**Design, Setting, and Participants:**

This repeated cross-sectional study used data from the Household Pulse Survey covering April 14, 2021, to January 10, 2022, in the US. A difference-in-difference-in-differences estimator combined with propensity score matching was used to estimate the association of the expanded CTC with mental health symptoms among households with income less than $35 000.

**Exposure:**

The monthly payment of expanded CTC from July 15 to December 15, 2021.

**Main Outcomes and Measures:**

Parental mental health was measured by analyzing depression and anxiety symptoms using logistic regression.

**Results:**

The weighted sample comprised 546 366 adults (mean [SD] age, 43.02 [14.54] years; 52.9% female). The most common education level was high school or less (36.0%), the highest frequency of household income distribution was $50 000 to $74 999 (16.1%), and the majority of the sample was employed (67.3%). The weighted mean (SD) number of children in the household was 0.92 (1.18). For the full sample, receiving expanded CTC benefits was associated with lower odds of experiencing anxiety symptoms (odds ratio, 0.730; 95% CI, 0.598-0.890). Subgroup analyses indicated that the positive associations of the policy with anxiety symptoms were particularly pronounced among the female, working-age (17-60 years), non-Hispanic White, and higher education groups. However, the policy expansion had no association with depression.

**Conclusions and Relevance:**

These findings may provide valuable evidence for policy makers to consider when deliberating on the possibility of making the CTC expansion permanent or transforming it into a universal program.

## Key Points

**Question:**

Was the expansion of the Child Tax Credit (CTC) in 2021 associated with improved mental health among parents with low income in US households?

**Findings:**

In this cross-sectional study of a weighted sample of 546 366 adults, the expanded CTC benefits were associated with an approximate one-fourth decrease in anxiety symptoms for the primary beneficiaries whose household income was less than $35 000.

**Meaning:**

These findings of improved parental anxiety symptoms with the CTC expansion may provide valuable evidence to inform policy makers’ consideration of making the CTC expansion permanent or transforming it into a universal program.

## Teaser

This cross-sectional study uses Household Pulse Survey data to investigate the association between the 2021 Child Tax Credit expansion and mental health among parents with low income.

## Introduction

Mental health is a pressing public health concern, serving as a fundamental pillar of both individual well-being and the broader health of society. The COVID-19 pandemic has substantially heightened concerns about mental health. Severe economic hardships from the widespread lockdowns and business closures, coupled with the fear of the virus, resulted in a substantial surge in the prevalence of mental health challenges.^1^ Moreover, the pandemic has unveiled disproportionate effects that vary based on social, economic, and personal circumstances.^2,3,4,5^

In July 2021, in response to the economic downturn caused by the pandemic, the US government temporarily expanded the Child Tax Credit (CTC), an income transfer program established in 1997 to provide financial aid for families with children, as part of the American Rescue Plan Act of 2021. Studies have found that relevant policies, such as the Temporary Assistance for Needy Families, the Supplemental Nutrition Assistance Program, and the Earned Income Tax Credit, may play pivotal roles in promoting mental health among families with low income through multiple channels.^6,7,8^ For instance, additional income reduces financial burden and material hardships. It also allows parents to invest in resources that promote family well-being and child development. Conversely, a scarcity of resources contributes to heightened stress levels, with detrimental outcomes for mental health. Additionally, resource constraints may impede effective parenting and strain family relationships, further exacerbating parental mental health issues.

The 2021 CTC expansion marked a pivotal transformation in US social policy. It raised the credit amount per child, expanded eligibility to include previously excluded parents with low income, and transitioned to a monthly advance payment system instead of lump sum disbursements. Prior to the expansion, the CTC was only partially refundable, leading to some families missing out on the full benefit. The changes took effect on July 15, 2021, with the Internal Revenue Service delivering advanced monthly installments of up to $250 per child aged 6 to 17 years and up to $300 per child younger than 6 years. This expansion aimed to provide financial relief and promote child development by making the credit nearly universal with more generous credit amounts and periodic distributions.^9,10^

While we know that the 2021 CTC expansion has been shown to improve various aspects of family well-being and economic outcomes,^2,9,10,11,12^ it is important to note that there are currently only a handful of relevant studies on the association of the policy with mental health. The aim of our study was to fill this crucial gap by identifying the association of the 2021 CTC expansion with parental mental health as measured by depression and anxiety symptoms using a refined methodology. Furthermore, we examined heterogenous policy outcomes across various demographic characteristics based on findings from prior studies^11,13,14^ in order to distinguish groups that may have been particularly vulnerable during the pandemic and investigate how individuals with different socioeconomic backgrounds responded to additional income from the policy change. Thus, we hypothesized that the policy expansion was associated with improved mental health among parents with low income.

## Methods

### Data

This cross-sectional study used data from the Household Pulse Survey (HPS) collected by the US Census Bureau in collaboration with federal agencies. The data collection started on April 23, 2020, with the primary objective of gathering nationally representative information on the experiences of US households during the pandemic. This study was exempt from review and informed consent under the Common Rule (45 CFR 46) given the use of secondary deidentified data. This study follows the Strengthening the Reporting of Observational Studies in Epidemiology (STROBE) reporting guideline for cross-sectional studies.

The HPS comprises a 20-minute online questionnaire. While the survey is limited in its relatively lower response rate than other federally sponsored surveys, it offers unique advantages in delivering timely and comprehensive information ranging from demographic characteristics to mental health indicators.^15^ The current study analyzes data spanning from April 14, 2021, to January 10, 2022 (weeks 28-41). The policy expansion, marked by the first monthly payment on July 15, 2021, allowed us to create 2 distinct periods: the pre–policy expansion period (weeks 28-33) and the post–policy expansion period (weeks 34-41). It should be noted that the monthly payments were delivered over 6 months from July 15 to December 15, 2021, and households received the remainder of their credit in a lump sum payment after filing their taxes in the spring of 2022.^16^ To mitigate potential confounding effects from variations in payment delivery methods, we focused our post–policy expansion period on July to December 2021.

### Measures

The main mental health outcomes of interest consisted of self-reported depression and anxiety symptoms. Depression in the HPS was measured using a modified version of the 2-item Patient Health Questionnaire. The 2 questions asked how often respondents have been bothered by having little interest or pleasure in doing things and by feeling down, depressed, or hopeless. Similarly, anxiety symptoms were assessed using the 2-item Generalized Anxiety Disorder scale. Respondents were asked about the frequency of feeling nervous, anxious, or on edge and by not being able to stop or control worrying. The response categories of both parts of the questionnaire included not at all, several days, more than half the days, and nearly every day, with scores ranging from 0 to 3. We combined the scores for depression and anxiety, respectively, and created 2 binary variables indicating a high risk of depression or anxiety for scores of 3 or higher.^17^

It should be noted that for the pre–policy expansion period, both depression and anxiety measures inquired about experiences over the past 7 days. However, in the post–policy expansion period, the questionnaire changed, and these measures now cover the last 2 weeks, potentially introducing measurement errors. To address the inconsistency in reference periods between the pre- and post-policy phases, we conducted a sensitivity test using alternative outcome measures. As the reference categories of the questions for depression and anxiety are based on the number of days (not at all, several days, more than half the days, or nearly every day), we adjusted these categories to a scale from 0 to 1, making the measures time insensitive. Specifically, we assigned 0 to not at all, while several days, more than half the days, and nearly every day were scored as 1. Subsequently, if the sum of the 2 questions for each symptom score was 2 or higher, we coded each depression and anxiety indicator as 1. The findings presented in eTable 4 in Supplement 1 indicate that the change in the reference period did not qualitatively change our findings, despite some inconsistencies in statistical significance in certain cases (ie, where the odds ratios [ORs] for depression were statistically significant in some cases, including the model for the full sample).

We used living with children in the households as a proxy for CTC treatment status. It is important to note that parents or guardians can still be eligible for the CTC even if they do not live with qualifying children under certain conditions. For instance, they may still qualify if they have lived with the children for more than half of the year. However, in this study, to ensure clarity and avoid complications related to family complexity and tax rules, we defined CTC eligibility based on the presence of resident children in the household. One methodological concern with our treatment variable is that there may be systematic differences between adults with children (eligible for the CTC) and those without (not eligible for the CTC). Such differences could potentially introduce bias. We address such differences by balancing these 2 groups using a propensity score matching (PSM) method, as discussed in the following section.

### Statistical Analysis

#### Propensity Score Matching

We matched adults living with children to those not living with children using a PSM method to minimize potential biases in the estimates of the association of the expanded CTC with mental health that may arise from imbalances between the 2 groups. Importantly, due to the cross-sectional design of the HPS, we cannot track the same observations longitudinally. In other words, it is not possible to match the 2 groups based on preexisting variables. In this context, we use Aerts and Schmidt’s^18^ multiple matching processes to convert cross-sectional data into quasi-panel data, enabling us to balance the treated and control groups using repeated cross-sectional data, as illustrated in the eFigure in Supplement 1. The following household characteristics were included to generate propensity scores: age, sex, self-reported race and ethnicity (Hispanic; non-Hispanic Black; non-Hispanic White; or non-Hispanic Asian, other race and ethnicity, or multiple races [grouped because of small sample sizes]), marital status, education (high school graduate or below, some college, and bachelor’s degree or higher), household income (<$25 000, $25 000-$34 999, $35 000-$49 999, $50 000-$74 999, $75 000-$99 999, $100 000-$149 999, $150 000-$199 999, or≥$200 000), and employment status. We included the race and ethnicity variable as prior studies showed that the pandemic had differential effects based on race and ethnicity. The public data did not disclose the races and ethnicities that comprised the other category because of small numbers. In the first matching stage (matching A), we matched individual *i* in the treated group (eligible for CTC) in the post–policy expansion period *t_1_* with a nontreated (not eligible for CTC) twin *h* in the same period. In this stage, we used a 1-to-1 nearest neighbor matching method without replacement. Subsequently, in the second matching stage (matching B), we used the matched samples from matching A to match CTC-eligible individuals in the post–policy expansion period with a twin *k* from the pre–policy expansion period *t_0_*. In the final matching stage (matching C), using matched samples from matching A, we matched non–CTC-eligible individuals in the post period with a twin *j* in the pre period. In matching B and C, we used a nearest neighbor matching method with replacement, allowing the control units to be matched to multiple treated units, thus preserving observations. These 3 stages of matching enabled us to create balanced samples across the treated and nontreated units and over time and to address potential biases in estimating the effects of the CTC policy expansion. eTable 1 in Supplement 1 illustrates the results from the PSM balance diagnostics. It shows that bias between the CTC-eligible and non–CTC-eligible groups was significantly reduced (by ≥90%) for most of the variables used in the PSM process.

#### Triple-Difference Model

This study examined the association of the expanded CTC with mental health using a difference-in-difference-in-differences, or triple-difference, model with PSM samples. We used a logistic regression method because our mental health measures were binary. The triple-difference model is constructed as follows:

logit(*Ŷ_iwst_*) = *α* + *β*_1_(*POST* · *CTC* · *POVERTY*)*_iwst_* + *β*_2_(*POST* · *CTC*)*_iwst_* + *β*_3_(*POST* · *POVERTY*)*_iwst_* + *β*_4_(*CTC* · *POVERTY*)*_iwst_* + *β*_5_*POST_iwst_* + *β*_6_*CTC_iwst_* + *β*_7_*POVERTY_iwst_* + *X_iwst_Φ* + *γ_w_* + *η_st_* + *e_iwst_*

where *Ŷ_iwst_* is the dependent variable for individual *i* surveyed in week *w* residing in state *st*. The *POST* variable is an indicator variable set to 1 for the post–policy expansion period after the first payment of the expanded CTC on July 15, 2021, and coded 0 before July 15. The *CTC* variable takes a value of 1 if individuals were living with CTC-qualifying children, which we used as a proxy for CTC eligibility, and coded 0 if otherwise. The *POVERTY* variable equals 1 if the household income was less than $35 000. This variable captures individuals who were potentially excluded from the CTC before the policy expansion and became newly eligible in response to the policy expansion in 2021. The triple-interaction term estimates the effects of the expanded CTC on mental health for the primary beneficiaries of the expansion. The *β_1_* variable is the coefficient of interest in this study. The *X* variable denotes all self-reported covariates that are potentially associated with the CTC eligibility status and mental health, including sex, age, race and ethnicity, marital status, education, household income, employment status, and number of children in the household. We include time dummies (*γ_w_*) and state dummies (*η_st_*) to account for time-varying trends that were consistent across individuals and heterogeneity between states, all of which may affect our main outcomes of interest. The Table presents weighted descriptive statistics for 4 groups, categorized by period and CTC eligibility, for the final analytic sample with nonmissing values on the outcome measures and key covariates.

**Table.** Descriptive Statistics^a^

	No. (weighted %)			
	Pre–policy expansion (before July 15, 2021)		Post–policy expansion (after July 15, 2021)	
	CTC-eligible (n = 16 608)	Non–CTC-eligible (n = 19 174)	CTC-eligible (n = 255 292)	Non–CTC-eligible (n = 255 292)
**Covariates**				
Age, mean (SD), y	44.48 (15.87)	44.43 (16.80)	43.41 (13.33)	42.38 (15.28)
No. of children in household, mean (SD)	1.83 (1.07)	0.00 (0.00)	1.86 (1.03)	0.00 (0.00)
Sex				
Female	9808 (49.6)	10 630 (46.3)	161 874 (54.6)	160 588 (52.1)
Male	6800 (50.4)	8544 (53.7)	93 418 (45.4)	94 704 (47.9)
Race and ethnicity				
Hispanic	3570 (29.6)	4011 (27.4)	29 674 (20.5)	27 640 (17.1)
Non-Hispanic Black	2687 (17.9)	3171 (18.1)	21 076 (12.3)	21 206 (11.1)
Non-Hispanic White	7592 (39.5)	8652 (40.6)	178 192 (57.0)	180 020 (61.4)
Non-Hispanic Asian, other race and ethnicity,^b^ or multiple races	2759 (13.0)	3340 (13.9)	26 350 (10.2)	26 426 (10.3)
Married	9104 (53.7)	9408 (47.6)	180 850 (64.7)	141 232 (51.6)
Education				
High school or less	3978 (52.8)	4064 (46.1)	31 304 (36.5)	31 682 (33.2)
Some college	6154 (29.5)	6999 (32.1)	76 226 (30.0)	78 728 (31.6)
Bachelor’s degree or higher	6476 (17.8)	8111 (21.8)	147 762 (33.5)	144 882 (35.2)
Annual income, $				
<25 000	2452 (18.5)	2919 (17.7)	24 066 (15.6)	27 866 (14.9)
25 000-34 999	2329 (17.7)	2529 (16.0)	19 012 (10.8)	21 442 (11.1)
35 000-49 999	2483 (15.4)	2689 (15.1)	23 062 (11.4)	25 856 (11.6)
50 000-74 999	2732 (16.9)	3082 (15.2)	37 290 (15.9)	40 184 (16.3)
75 000-99 999	2219 (11.4)	2457 (11.8)	34 976 (12.6)	34 078 (12.8)
100 000-149 999	1993 (9.8)	2442 (11.7)	51 992 (16.2)	47 612 (16.4)
150 000-199 999	1231 (5.2)	1574 (6.8)	27 702 (7.8)	25 178 (7.9)
≥200 000	1169 (5.1)	1482 (5.8)	37 192 (9.6)	33 076 (9.1)
Employed	9130 (54.6)	10 738 (57.8)	184 618 (66.0)	190 454 (70.6)
**Mental health outcomes**				
Depression	3921 (26.9)	4531 (27.7)	50 794 (23.2)	56 936 (27.0)
Anxiety	4994 (32.3)	5393 (30.6)	75 188 (31.4)	72 846 (31.7)

Abbreviation: CTC, Child Tax Credit.

^a^ The source is our own analyses of data from the Household Pulse Survey, April 14, 2021, to January 10, 2022.^15^ Sample weights were applied.

^b^ The other category reflects all races and ethnicities available in the public use data, which was not expanded further due to small numbers.

## Results

Our weighted sample comprised 546 366 adults with a mean (SD) age of 43.02 (14.54) years. More than one-half of the sample were female (52.9% compared with 47.1% male) and non-Hispanic White (57.7% compared 12.2% non-Hispanic Black and 10.5% non-Hispanic Asian, other race and ethnicity, or multiple races), and Hispanic individuals accounted for 19.6% of the sample. The most common education level was high school graduate or less (36.0%). Regarding income distribution, the highest frequency was observed in the $50 000 to $74 999 range (16.1%), followed by $100 000 to $149 999 (15.9%), and less than $25 000 (15.5%). The majority of the sample was employed (67.3%). The weighted mean (SD) number of children in the household was 0.92 (1.18).

The Figure shows the estimated ORs and the 95% CIs from the triple-difference model with PSM samples for the full sample and for subgroups defined by demographic characteristics, including sex, age, race and ethnicity, marital status, and education. The ORs were derived from the triple-interaction term, representing the estimated associations of the expanded CTC with mental health for the primary beneficiaries with household incomes below $35 000. The CTC expansion was not significantly associated with a change in depression (OR, 0.857; 95% CI, 0.694-1.059). The OR for anxiety symptoms for the full sample was 0.730 (95% CI, 0.598-0.890), implying a significant reduction in anxiety symptoms associated with the CTC expansion. Our findings for the full sample suggest that the expanded CTC benefits delivered from July to December 2021 may have contributed to a nearly one-fourth decrease in anxiety symptoms for the primary beneficiaries whose household income was less than $35 000.

**Figure.**
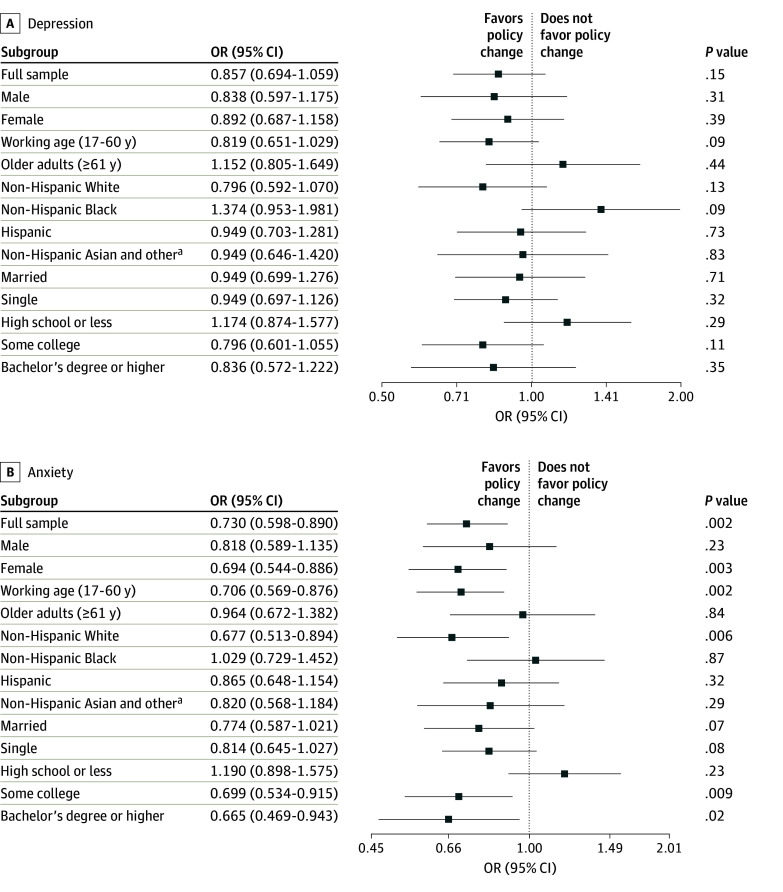
Outcomes of the Child Tax Credit (CTC) Expansion in 2021 on Mental Health Among Parents With Low Income, April 2021 to January 2022 The source of these findings is our analyses of data from the Household Pulse Survey, April 14, 2021, to January 10, 2022.^15^ Odds ratios (ORs) are plotted as point estimates with 95% CIs. The ORs are derived from triple-difference models in which the primary exposure is a triple-interaction term between a binary variable representing that the interview was conducted after the CTC expansion (July 15, 2021), an indicator for CTC eligibility, and a binary variable for whether household income was below $35 000. All logistic regressions are adjusted for sex, age, race and ethnicity, marital status, income, education, employment status, and number of children in the household. Biweekly fixed effects and state fixed effects also were accounted for. Robust SEs were applied in the logistic regression models. ^a^The other category reflects all races and ethnicities available in the public use data, which was not expanded further due to small numbers.

Next, we performed a set of subgroup analyses by demographic characteristics using separate regression models for each subgroup. The subgroup analysis by sex showed that the OR for anxiety symptoms among female respondents was statistically significant (0.694; 95% CI, 0.544-0.886), suggesting a favorable association of the policy with anxiety symptoms for the female group. However, no association with mental health was observed among male respondents. We further stratified the full PSM sample based on age into 2 groups: working age (17-60 years) and older adults (≥61 years). The OR for anxiety symptoms for the working-age group was statistically significant (0.706; 95% CI, 0.569-0.876), while the OR for the same measure for older adults was not (0.964; 95% CI, 0.672-1.382). Again, the ORs for depression were consistently not significant for both age groups. In the subsequent subgroup analysis by race and ethnicity, the expanded CTC benefits did not show an association with any of the mental health measures for any of the racial and ethnic groups examined with the exception of anxiety symptoms among non-Hispanic White respondents (OR, 0.677; 95% CI, 0.513-0.894), which was an almost one-third improvement. The next subgroup analysis by marital status showed that the extended CTC benefits were not associated with mental health measures for either married or single parents. Finally, the subgroup analysis based on education level (high school or less, some college, and bachelor’s degree or higher) showed that the policy expansion was associated with a decrease in anxiety symptoms for the some college (OR, 0.699; 95% CI, 0.534-0.915) and bachelor’s degree or higher (OR, 0.665; 95% CI, 0.469-0.943) groups.

As a robustness check, we also estimated the regression models by ordinary least squares (eTable 2 in Supplement 1), which were qualitatively consistent with the main findings from the logistic regression models, and performed estimations using the raw sample before PSM (eTable 3 in Supplement 1), which supported that the results derived from the PSM sample were more favorable. Furthermore, changing the reference period did not qualitative change the findings, as discussed in the Measures section (eTable 4 in Supplement 1).

In summary, our analyses highlight that, on average, the extended CTC benefits were associated with an improvement in anxiety symptoms among parents with low income. The subgroup analyses indicated that the positive associations of the policy with anxiety symptoms were particularly pronounced among the female, working-age, non-Hispanic White, some college, and bachelor’s degree or higher subgroups. However, the policy expansion had null associations with depression.

## Discussion

In this cross-sectional study, we investigated the association of the 2021 CTC expansion with mental health outcomes among parents with low income, with a specific focus on depression and anxiety symptoms. We found that the expanded CTC was associated with alleviating parental anxiety symptoms. However, distinct variations existed within specific segments of the population.

In general, our findings align with several prior studies that indicated that the expanded CTC had favorable associations with alleviating mental health outcomes, including depression and anxiety symptoms,^11,13^ while diverging from those of Glasner et al,^14^ who reported no short-term association of the CTC expansion with measures of life satisfaction and anxiety and depression symptoms. Given that several studies have shown that the expanded CTC significantly improved financial burdens and economic difficulties, including poverty and material hardship,^10,12,19^ all of which are strongly associated with mental health, it is plausible to posit that the positive outcomes on these economic aspects may have partially or entirely translated into enhancements in parental mental health, as evidenced in the present study.

The findings from the subgroup analyses indicate that less disadvantaged groups experienced notable improvements in mental health outcomes. For example, working-age, non-Hispanic White, and more highly educated parents experienced a reduction in anxiety symptoms. In contrast, relatively more disadvantaged groups, including parents from racial and ethnic minority groups, older adults, and parents with less education, did not exhibit any observable improvements in their mental health. These findings raise the possibility that these groups may have been excluded from fully experiencing the advantages of the policy expansion. Furthermore, considering the disproportionate negative effects of the pandemic and the heightened challenges faced by more marginalized populations,^4,20,21^ these results show the intersectional outcomes of the pandemic. In other words, the intersections of multiple aspects of inequalities, specifically age, race and ethnicity, and education, within our sample experiencing poverty may result in compounded outcomes when individuals experience multiple disadvantaged positions.^22^ Thus, the CTC expansion, on its own, may not suffice in addressing the broader mental health issues that could be more pronounced among these highly vulnerable demographics.

Even more intriguingly, our findings differ in several ways from those of previous studies^11,13^ that used the same data from the HPS. First, our study found that the expanded CTC was associated with an improvement solely in anxiety symptoms, not depressive symptoms. In contrast, Batra et al^11^ and Cha et al^13^ reported that the policy expansion was associated with an enhancement in both symptoms. Second, these prior studies reported more pronounced positive policy associations among racial and ethnic minority groups. Such a disparity in results from the subgroup analyses underscores the need for more rigorous methodological approaches to identify the policy associations. The distinctiveness of our results may be attributable to the methodological rigor we have meticulously integrated. To be more specific, by using the PSM technique, the current study achieved better balance between the treated and untreated groups, accounting for heterogeneity between the 2 groups in estimating the true outcomes of the CTC expansion. This enhanced methodological approach instills higher confidence in the reliability and validity of our findings, setting our research apart from previous research. In addition, we provide in eTable 3 in Supplement 1 estimations using the raw sample before the PSM. The results from the raw sample indicate that the estimates derived from the PSM sample are more favorable, reinforcing the validity of our use of PSM.

### Limitations

The current study is not without limitations. First, the HPS data may be susceptible to a high rate of nonresponse bias,^15^ potentially leading to underrepresentation of the US population.^15,23,24,25^ The national-level weighted response rates from weeks 28 to 41 range from 5.4% to 7.4%, according to the HPS technical documentation for phases 3.1 to 3.3.^26^ Consequently, we emphasize the need for careful interpretation of the results, especially for subgroups, as quality matters more than quantity.^23^ Nevertheless, it is important to highlight that Parolin et al^27^ found that the HPS sample closely mirrors the Current Population Survey population estimates. Second, the literature generally suggests that people with better health may be more likely to participate voluntarily in health surveys.^28^ This inclination may lead to an underestimation of mental health issues, especially among individuals at higher risk. Therefore, the combination of low response rates and the self-reported nature of mental health data requires caution in interpreting the results. Third, our model only considers the number of children eligible for the CTC. The benefit levels of the expanded CTC differed based on the age of the child, providing a larger amount for younger children up to age 6 years. However, due to the data availability issue, we were unable to incorporate the number of children by age in the model.

## Conclusions

Although the 2021 CTC expansion held the potential to enhance economic outcomes,^10,12,19^ which in turn may have led to improved mental health as our findings suggest, it was temporary and expired by the end of 2021. Reverting the program to its former state was expected to result in an immediate reduction in the benefit level, which could potentially exacerbate parental mental health conditions and child poverty.^13^ Consequently, the expiration of the program raised substantial public health and economic concerns about its potential negative impact on the well-being of vulnerable populations, underscoring the need for continued support and comprehensive policy solutions, particularly as economic recovery from the pandemic prolongs and continues to place additional strain on vulnerable populations. Debates continue regarding the program’s permanence, alongside federal- and state-level consideration for similar programs. In fact, there are ongoing political efforts to revive the policy expansion, as evidenced by President Biden’s 2024 fiscal year budget proposal for the government^29^ and the introduction of the American Family Act of 2023 by Representatives Rosa DeLauro, Suzan DelBene, and Ritchie Torres. In this context, our findings may provide valuable evidence for policy makers and offer essential insights to inform decision making and shape future policies.

## Notes

Supplement 1. **eFigure.** Propensity Score Matching Methodology **eTable 1.** Propensity Score Matching Balance Diagnostics **eTable 2.** Effect of the CTC Expansion in 2021 on Mental Health Among Low-Income Parents Estimated From the OLS Model, April 2021-January 2022 **eTable 3.** Effects of the CTC Expansion in 2021 on Mental Health Among Low-Income Parents Using the Non-PSM Sample, April 2021-January 2022 **eTable 4.** Sensitivity Test: Effects of the CTC Expansion in 2021 on Mental Health Among Low-Income Parents Using Alternative Mental Health Measures, April 2021-January 2022

Supplement 2.
Data Sharing Statement
