# Supplementary material for: Expansion of Child Tax Credits and Mental Health of Parents With Low Income in 2021
Source: JAMA Netw Open. 2024 Feb 21;7(2):e2356419. doi: 10.1001/jamanetworkopen.2023.56419 (PMC10882416; doi:10.1001/jamanetworkopen.2023.56419)
Supplement: Supplement 1. — eFigure. Propensity Score Matching Methodology eTable 1. Propensity Score Matching Balance Diagnostics eTable 2. Effect of the CTC Expansion in 2021 on Mental Health Among Low-Income Parents Estimated From the OLS Model, April 2021-January 2022 eTable 3. Effects of the CTC Expansion in 2021 on Mental Health Among Low-Income Parents Using the Non-PSM Sample, April 2021-January 2022 eTable 4. Sensitivity Test: Effects of the CTC Expansion in 2021 on Mental Health Among Low-Income Parents Using Alternative Mental Health Measures, April 2021-January 2022 [file jamanetwopen-e2356419-s001.pdf]

Supplemental Online Content

Nam J, Kwon SJ. Expansion of Child Tax Credits and mental health of parents with low income in 2021. *JAMA Netw Open*. 2024;7(2):e2356419. doi:10.1001/jamanetworkopen.2023.56419

eFigure. Propensity Score Matching Methodology

eTable 1. Propensity Score Matching Balance Diagnostics

eTable 2. Effect of the CTC Expansion in 2021 on Mental Health Among Low-Income Parents Estimated From the OLS Model, April 2021-January 2022

eTable 3. Effects of the CTC Expansion in 2021 on Mental Health Among Low-Income Parents Using the Non-PSM Sample, April 2021-January 2022

eTable 4. Sensitivity Test: Effects of the CTC Expansion in 2021 on Mental Health Among Low-Income Parents Using Alternative Mental Health Measures, April 2021-January 2022

This supplemental material has been provided by the authors to give readers additional information about their work.

© 2024 Nam J et al. *JAMA Network Open.*

eFigure. Propensity Score Matching Methodology

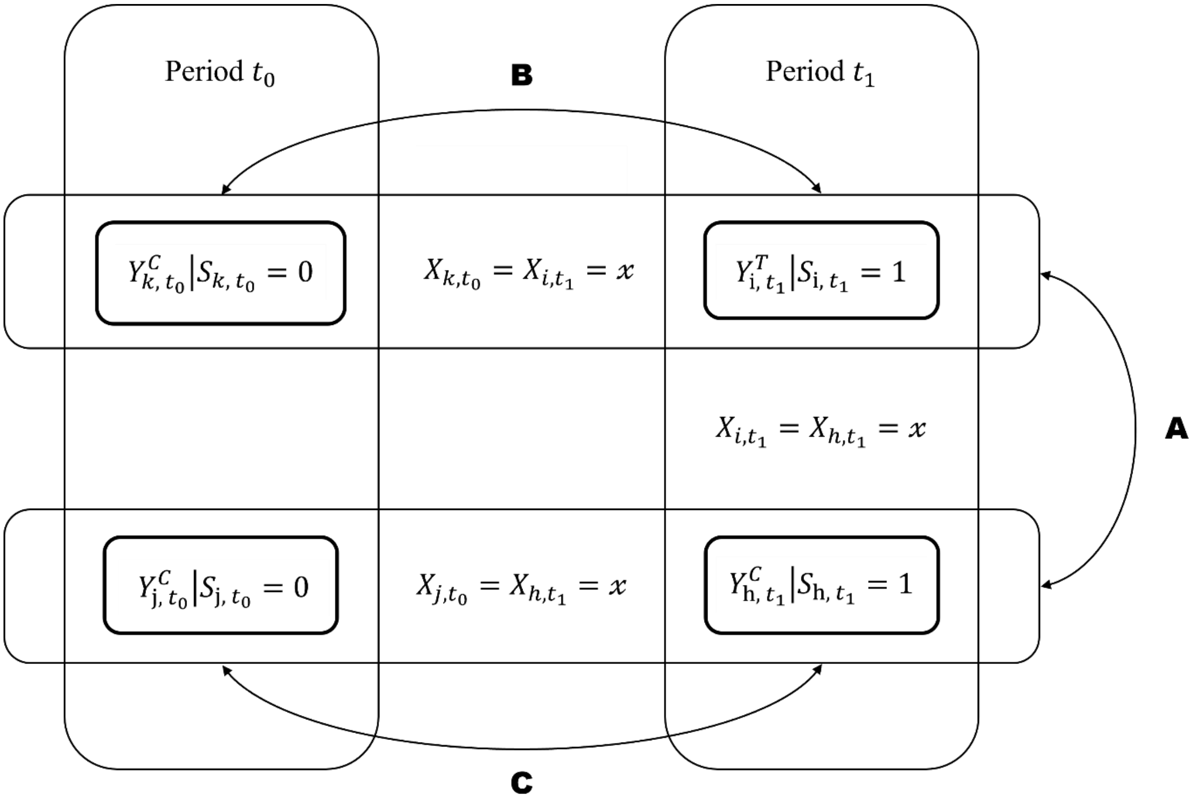

Source: Aerts & Schmidt.[16(p811)]

© 2024 Nam J et al. *JAMA Network Open.*

eTable 1. Propensity Score Matching Balance Diagnostics

Variables	Sample U (Unmatched) / M (Matched)	Mean CTC-eligible	Mean Non-CTC-eligible	% bias	% reduction in bias	t-test t	t-test p-value
Age	U	45.31	49.30	-27.4	98.7	-54.74	0.000
	M	45.31	45.25	0.4		1.65	0.099
Female	U	0.63	0.57	12.4	92.1	22.85	0.000
	M	0.63	0.64	-1		-4.06	0.000
Black	U	0.08	0.16	-24.8	96.9	-52.32	0.000
	M	0.08	0.08	0.8		3.82	0.000
Hispanic	U	0.11	0.21	-27.3	96.3	-56.47	0.000
	M	0.11	0.11	1		4.85	0.000
Asian and other	U	0.10	0.17	-19.6	93.1	-39.62	0.000
	M	0.10	0.10	1.3		6.27	0.000
Married	U	0.63	0.52	23.1	99.9	42.89	0.000
	M	0.63	0.63	0		-0.14	0.892
Some college	U	0.30	0.37	-13.6	99.9	-25.41	0.000
	M	0.30	0.30	0		0.05	0.962
BA or above	U	0.57	0.41	33.6	98.1	61.21	0.000
	M	0.57	0.58	-0.6		-2.65	0.008
$25,000-$34,999	U	0.08	0.14	-18.3	96.9	-37.54	0.000
	M	0.08	0.08	0.6		2.66	0.008
$35,000-$49,999	U	0.10	0.14	-15	96.7	-29.86	0.000
	M	0.10	0.10	0.5		2.23	0.025
$50,000-$74,999	U	0.15	0.16	-3	92.7	-5.47	0.000
	M	0.15	0.15	-0.2		-0.91	0.365
$75,000-$99,999	U	0.14	0.13	1.3	72.3	2.44	0.015
	M	0.14	0.14	-0.4		-1.52	0.127
$100,000-$149,999	U	0.20	0.13	19.5	97.4	33.16	0.000
	M	0.20	0.20	-0.5		-1.95	0.051
$150,000-$199,999	U	0.10	0.08	8.8	98.8	15.22	0.000
	M	0.10	0.10	-0.1		-0.41	0.679
$200,000 and above	U	0.14	0.08	20.8	98.7	34.20	0.000
	M	0.14	0.14	0.3		1.00	0.316
Employed	U	0.73	0.56	38.2	96.3	73.63	0.000
	M	0.73	0.74	-1.4		-6.16	0.000

Source: Authors' own analyses of data from the Household Pulse Survey, April 14, 2021 to January 10, 2022.

© 2024 Nam J et al. *JAMA Network Open.*

eTable 2. Effects of the CTC Expansion in 2021 on Mental Health Among Low-Income Parents Estimated From the OLS Model, April 2021-January 2022

	Depression		Anxiety	
	Coefficient	95% CI	Coefficient	95% CI
**Panel 1. Full sample**				
Full sample	-0.030	-0.066, 0.007	-0.061**	-0.100, -0.021
N	546,366		546,366	
**Panel 2. By sex**				
Male	-0.025	-0.078, 0.029	-0.033	-0.091, 0.025
N	203,466		203,466	
Female	-0.025	-0.072, 0.021	-0.075**	-0.125, -0.024
N	342,900		342,900	
**Panel 3. By age**				
Non-elderly	-0.042*	-0.083, -0.000	-0.071**	-0.115, -0.026
N	473,401		473,401	
Elderly	0.034	-0.020, 0.088	0.007	-0.052, 0.067
N	72,965		72,965	
**Panel 4. By race/ethnicity**				
Non-Hispanic White	-0.042	-0.094, 0.010	-0.074**	-0.129, -0.018
N	374,456		374,456	
Non-Hispanic Black	0.047	-0.021, 0.115	0.010	-0.062, 0.081
N	48,140		48,140	
Hispanic	-0.010	-0.070, 0.050	-0.036	-0.099, 0.027
N	64,895		64,895	
Non-Hispanic Asian and any other race alone, or race in combination	-0.014	-0.086, 0.058	-0.038	-0.113, 0.036
N	58,875		58,875	
**Panel 5. By marital status**				
Married	-0.002	-0.050, 0.047	-0.039	-0.092, 0.014
N	340,594		340,594	
Single	-0.030	-0.078, 0.018	-0.047	-0.097, 0.004
N	205,772		205,772	
**Panel 6. By education level**				
High School or below	0.025	-0.032, 0.081	0.034	-0.024, 0.092
N	71,028		71,028	
Some college	-0.043	-0.099, 0.014	-0.073*	-0.131, -0.015
N	168,107		168,107	
BA or above	-0.033	-0.095, 0.030	-0.077*	-0.146, -0.008
N	307,231		307,231	

Source: Authors' own analyses of data from the Household Pulse Survey, April 14, 2021 to January 10, 2022.
Notes: CI = confidence interval. Robust standard errors are used. Coefficients are derived from triple-difference models in which the primary exposure is a triple-interaction term between a binary variable representing that the interview was conducted after the CTC expansion (July 15, 2021), an indicator for CTC-eligibility, and a binary variable for whether household income is below $35,000. All OLS regressions adjusted for sex, age, race and ethnicity, marital status, income, education, employment status and number of children in the household. Additionally, biweekly fixed effects and state fixed effects were accounted for. **$p < 0.01$, *$p < 0.05$

© 2024 Nam J et al. *JAMA Network Open.*

eTable 3. Effects of the CTC Expansion in 2021 on Mental Health Among Low-Income Parents Using the Non-PSM Sample, April 2021-January 2022

	Depression		Anxiety	
	Odds Ratio	95% CI	Odds Ratio	95% CI
**Panel 1. Full sample**				
Full sample	1.023	0.916, 1.143	0.912	0.820, 1.014
N	546,366		546,366	
**Panel 2. By sex**				
Male	1.144	0.953, 1.373	1.134	0.951, 1.351
N	203,466		203,466	
Female	1.008	0.875, 1.160	0.837**	0.732, 0.957
N	342,900		342,900	
**Panel 3. By age**				
Non-elderly	0.959	0.849, 1.083	0.837**	0.745, 0.940
N	473,401		473,401	
Elderly	0.893	0.683, 1.166	0.888	0.691, 1.141
N	72,965		72,965	
**Panel 4. By race/ethnicity**				
Non-Hispanic White	1.009	0.855, 1.191	0.947	0.808, 1.110
N	374,456		374,456	
Non-Hispanic Black	1.133	0.855, 1.502	1.004	0.770, 1.310
N	48,140		48,140	
Hispanic	0.965	0.763, 1.222	0.863	0.689, 1.080
N	64,895		64,895	
Non-Hispanic Asian and any other race alone, or race in combination	1.006	0.760, 1.333	0.848	0.647, 1.110
N	58,875		58,875	
**Panel 5. By marital status**				
Married	1.036	0.870, 1.233	0.879	0.745, 1.037
N	340,594		340,594	
Single	0.969	0.837, 1.123	0.907	0.786, 1.046
N	205,772		205,772	
**Panel 6. By education level**				
High School or below	0.920	0.740, 1.142	0.945	0.768, 1.163
N	71,028		71,028	
Some college	1.073	0.900, 1.279	0.934	0.787, 1.108
N	168,107		168,107	
BA or above	1.178	0.960, 1.444	0.961	0.793, 1.166
N	307,231		307,231	

Source: Authors' own analyses of data from the Household Pulse Survey, April 14, 2021 to January 10, 2022.
Notes: CI = confidence interval. Robust standard errors are used. Odds ratios are derived from triple-difference models in which the primary exposure is a triple-interaction term between a binary variable representing that the interview was conducted after the CTC expansion (July 15, 2021), an indicator for CTC-eligibility, and a binary variable for whether household income is below $35,000. All logistic regressions adjusted for sex, age, race and ethnicity, marital status, income, education, employment status and number of children in the household. Additionally, biweekly fixed effects and state fixed effects were accounted for. **$p < 0.01$, *$p < 0.05$

© 2024 Nam J et al. *JAMA Network Open.*

eTable 4. Sensitivity Test: Effects of the CTC Expansion in 2021 on Mental Health Among Low-Income Parents Using Alternative Mental Health Measures, April 2021-January 2022

	Depression		Anxiety	
	Odds Ratio	95% CI	Odds Ratio	95% CI
**Panel 1. Full sample**				
Full sample	0.764**	0.637, 0.918	0.729**	0.608, 0.874
N	546,366		546,366	
**Panel 2. By sex**				
Male	0.798	0.603, 1.058	0.770	0.582, 1.018
N	203,466		203,466	
Female	0.764*	0.607, 0.963	0.738*	0.586, 0.930
N	342,900		342,900	
**Panel 3. By age**				
Non-elderly	0.729**	0.593, 0.896	0.699**	0.569, 0.859
N	473,401		473,401	
Elderly	0.935	0.691, 1.265	0.901	0.665, 1.220
N	72,965		72,965	
**Panel 4. By race/ethnicity**				
Non-Hispanic White	0.762*	0.586, 0.990	0.695**	0.536, 0.902
N	374,456		374,456	
Non-Hispanic Black	0.836	0.604, 1.156	0.799	0.578, 1.103
N	48,140		48,140	
Hispanic	0.795	0.602, 1.050	0.963	0.728, 1.273
N	64,895		64,895	
Non-Hispanic Asian and any other race alone, or race in combination	0.806	0.562, 1.157	0.744	0.517, 1.071
N	58,875		58,875	
**Panel 5. By marital status**				
Married	0.889	0.689, 1.146	0.842	0.656, 1.082
N	340,594		340,594	
Single	0.693**	0.555, 0.866	0.708**	0.566, 0.886
N	205,772		205,772	
**Panel 6. By education level**				
High School or below	0.996	0.759, 1.308	1.046	0.800, 1.367
N	71,028		71,028	
Some college	0.648**	0.500, 0.840	0.721*	0.552, 0.942
N	168,107		168,107	
BA or above	0.828	0.599, 1.143	0.645**	0.472, 0.883
N	307,231		307,231	

Source: Authors' own analyses of data from the Household Pulse Survey, April 14, 2021 to January 10, 2022.
Notes: CI = confidence interval. Robust standard errors are used. Odds ratios are derived from triple-difference models in which the primary exposure is a triple-interaction term between a binary variable representing that the interview was conducted after the CTC expansion (July 15, 2021), an indicator for CTC-eligibility, and a binary variable for whether household income is below $35,000. All logistic regressions adjusted for sex, age, race and ethnicity, marital status, income, education, employment status and number of children in the household. Additionally, biweekly fixed effects and state fixed effects were accounted for. $^{**}p < 0.01$, $^{*}p < 0.05$

© 2024 Nam J et al. *JAMA Network Open.*
