# Supplementary material for: Expansion of Child Tax Credits and Mental Health of Parents With Low Income in 2021
Source: JAMA Netw Open. 2024 Feb 21;7(2):e2356419. doi: 10.1001/jamanetworkopen.2023.56419 (PMC10882416; doi:10.1001/jamanetworkopen.2023.56419)
Supplement: Supplement 2. — Data Sharing Statement [file jamanetwopen-e2356419-s002.pdf]

Data Sharing Statement

Nam. Expansion of Child Tax Credits and Mental Health of Parents With Low Income in 2021. *JAMA Netw Open*. Published February 21, 2024. doi:10.1001/jamanetworkopen.2023.56419

Data
Data available: No
